# Harnessing data science to control non-communicable diseases in Africa: a systematic review and gap analysis

**DOI:** 10.1038/s43856-025-01272-0

**Published:** 2026-07-16

**Authors:** Akinkunmi Paul Okekunle, Osahon Jeffery Asowata, Adekunle Gregory Fakunle, Olasubomi Jimmy Omoleye, Muideen Tunbosun Olaiya, Judit Kumuthini, Moustafa I. Morsy, Elisabetta Caiazzo, Alison Annet Kinengyere, Grace Ajuwon, Elaine O. Nsoesie, Paul Olowoyo, Andre Pascal Kengne, Obaro S. Michael, Fred Stephen Sarfo, Folakemi T. Odedina, Segun Fatumo, Abiodun M. Adeoye, Akindele Olupelumi Adebiyi, Rufus Olusola Akinyemi, Charles Agyemang, Shakuntala Baichoo, Bruce Ovbiagele, Pasquale Maffia, Olufunmilayo Olopade, Benjamin Aribisala, Onoja Matthew Akpa, Yosr Hamdi, Mayowa Ojo Owolabi

**Affiliations:** 1 Department of Medicine, Faculty of Clinical Sciences, University of Ibadan, 200284 Ibadan, Nigeria; 2 Research Institute of Human Ecology, Seoul National University, 08826 Seoul, Republic of Korea; 3 Department of Epidemiology and Medical Statistics, University of Ibadan, 200284 Ibadan, Nigeria; 4 Department of Pharmacology and Toxicology, East Carolina University, North Carolina, USA; 5 Department of Public Health, Osun State University, Osogbo, Nigeria; 6 Department of Radiology, University of Chicago, Chicago, IL USA; 7 Department of Medicine, School of Clinical Sciences at Monash Health, Monash University, Clayton, VIC Australia; 8 South African National Bioinformatics Institute, University of Western Cape, Cape Town, South Africa; 9 School of Infection & Immunity, College of Medical, Veterinary and Life Sciences, University of Glasgow, Glasgow, UK; 10 Department of Pharmacy, School of Medicine and Surgery, University of Naples Federico II, Naples, Italy; 11 Sir Albert Cook Library, College of Health Sciences, Makerere University, Kampala, Uganda; 12 E. Latunde Odeku Medical Library, College of Medicine, University of Ibadan, 200284 Ibadan, Nigeria; 13 Department of Global Health, School of Public Health, Boston University, Boston, MA USA; 14 Department of Medicine, Federal Teaching Hospital, Ido–Ekiti, Nigeria; 15 Afe Babalola University, Ado-Ekiti, Nigeria; 16 South African Medical Research Council, Cape Town, South Africa; 17 Department of Pharmacology and Therapeutics, University of Ibadan, 200284 Ibadan, Nigeria; 18 Department of Medicine, Kwame Nkrumah University of Science and Technology, Kumasi, Ghana; 19 Cancer Prevention, Survivorship and Care Delivery (CPSCD) Research Program, Mayo Clinic Comprehensive Cancer Centre, Jacksonville, Florida USA; 20 Prostate Cancer Transatlantic Consortium (CaPTC), Jacksonville, Florida USA; 21 Precision Healthcare Research Institute, Queen Mary University of London, London, E1 4NS United Kingdom; 22 MRC/UVRI and LSHTM Uganda Research Unit, Entebbe, Uganda; 23 Department of Medicine, University College Hospital, Ibadan, 200285 Ibadan, Nigeria; 24 Institute of Cardiovascular Diseases, University of Ibadan, 200284 Ibadan, Nigeria; 25 College of Medicine, University of Ibadan, 200284 Ibadan, Nigeria; 26 Centre for Genomic and Precision Medicine, University of Ibadan, Ibadan, Nigeria; 27 Neuroscience and Ageing Research Unit, Institute for Advanced Medical Research and Training, College of Medicine, University of Ibadan, 200284 Ibadan, Nigeria; 28 Department of Public and Occupational Health, Amsterdam University Medical Centres, University of Amsterdam, 1105AZ Amsterdam, The Netherlands; 29 Department of Digital Technologies, FoICDT, University of Mauritius, Reduit, Mauritius; 30 Weill Institute for Neurosciences, University of California, San Francisco, USA; 31 Africa-Europe CoRE in Non-Communicable Diseases & Multimorbidity, African Research Universities Alliance (ARUA) & The Guild of European Research-intensive Universities, Glasgow, UK; 32 Centre for Innovation in Global Health, University of Chicago, Chicago, IL USA; 33 Department of Medicine, University of Chicago, Chicago, USA; 34 Department of Computer Science, Lagos State University, Ikeja, Nigeria; 35 Division of Epidemiology, Biostatistics and Environmental Health, School of Public Health, University of Memphis, Memphis, USA; 36 Laboratory of Biomedical Genomics and Oncogenetics, Institut Pasteur de Tunis, University of Tunis, El Manar, Tunis, Tunisia; 37 Blossom Specialist Medical Centre, Ibadan, Nigeria

**Keywords:** Public health, Medical research

## Abstract

**Background:**

Data science methods can provide novel and pragmatic approaches for preventing and controlling non-communicable diseases (NCDs) in Africa. This study highlights current efforts, opportunities, and challenges in leveraging data science methods to accelerate and advance the prevention and control of NCDs in Africa.

**Methods:**

We undertake a systematic review and gap analysis, as registered in PROSPERO (CRD42023406237).

**Results:**

Our findings suggest several data science methods have been used in research across the four leading NCDs in Africa. However, limited information exists on their application to improve disease surveillance, risk factor identification and characterization, prevention, treatment, drug discovery and rehabilitation. Machine learning outperforms traditional statistical methods in improving risk stratification in most studies (80.8%) designed for the prevention and control of NCDs. Notwithstanding, most (76.0%) data science techniques for NCDs prevention and control remain in the exploratory research phase, with limited clinical or public health application and minimal impact on the African population. There are critical gaps along the continuum of data generation, data quality, method development, and validation, which may be attributed to inadequate funding, capacity development, policy shortcomings, and infrastructure deficits. Considerable gaps exist in intra-African collaboration, data sharing, and replication, which hinder the cross-cultural replication and applicability of data science methods for NCDs prevention and control in Africa.

**Conclusions:**

Multi-sectoral interventions that promote interdisciplinary capacity building, investment, and knowledge linkages, taking into account indigenous epistemologies, are needed to harness the enormous potential of data science to accelerate the prevention and control of NCDs in Africa.

## Plain Language Summary

Data science offers innovative and practical approaches for accelerating the prevention and control of non-communicable diseases (NCDs). However, there are significant limitations that hinder their use for improving health outcomes in Africa. We highlight current efforts, opportunities, and challenges in leveraging data science to accelerate the prevention and control of NCDs in Africa. Most data science applications for NCD prevention and control in Africa remain in the exploratory phase, thereby limiting real-world clinical or public health utility. Multi-sectoral interventions that promote interdisciplinary capacity building, investment, and the linking of datasets are needed to harness the enormous potential of data science to accelerate the prevention and control of NCDs in Africa.

## Abstract (web summary)

Okekunle et al. assess the current trends of data science methods applied for non-communicable disease (NCD) prevention and control. Most data science applications for NCDs in Africa remain in the exploratory phase, with limited clinical or public health utility, thereby necessitating multisectoral interventions to harness their benefits.

## Introduction

In Africa, the burden of non-communicable diseases (NCDs), including cardiovascular diseases (CVDs), metabolic conditions, cancers, and chronic respiratory diseases, is increasing, overtaking infectious diseases^1^. The pathophysiology of NCDs involves complex interactions of genetic^2^, behavioral/lifestyle (e.g., diet, smoking, alcohol intake, and physical activity)^3^, and environmental (e.g., air pollution, climate change and work environment) factors^4–6^. In Africa, these complex interactions are also shaped by unique socio-economic factors, such as poverty and access to healthcare^7,8^. Therefore, novel methods are required to unravel these multifaceted interactions to inform the development of pragmatic interventions for the prevention and control of NCDs on the continent.

The growing availability of health information and biomedical data, coupled with advances in high-throughput analytic methods and tools^9,10^, has enhanced the application of data science for detailed investigation of the origins, pathophysiology, determinants, and outcomes of NCDs^11–13^. Data science involves developing and applying quantitative and analytical approaches, processes, and systems for large and complex datasets to provide knowledge and insights^14,15^, to improve health outcomes, especially in biomedical research, which takes into account the entire value chain of data science, including collection, processing, harmonization, analysis, visualization, interpretation, and reporting. The output of these processes helps develop pragmatic solutions for improving the prevention, diagnosis, prognosis, treatment, and control of diseases^16–20^.

While the application of data science in biomedical research is evolving in Africa^21^, little is known about its application for the prevention or control of NCDs in the region. For instance, it is unclear whether data science methods (DSM) add value more than traditional methods in enhancing the validity and utility of findings for NCDs prediction, diagnosis, prevention, and control. In addition, it is crucial to characterise knowledge gaps to identify emerging priorities and opportunities for generating evidence to inform data science solutions aimed at reducing the burden of NCDs in the region^22–24^.

This state-of-the-art systematic review and gap analysis was designed to assess the current trends, benefits, challenges, and opportunities of leveraging data science methods for the prevention and control of NCDs. Specifically, the work focused on four main NCDs (CVD, including stroke, cancer, type 2 diabetes, and chronic respiratory diseases) contributing to the most significant burden of diseases in Africa^1,25^ within the 4×4 framework of NCD health research and programming^26^, thereby proposing relevant recommendations to harness the potential of data science for the prevention and control of NCDs in Africa.

Data science methods offer novel and pragmatic approaches for accelerating the prevention and control of NCDs, but there are significant limitations that hinder harnessing their benefits to improve health outcomes in Africa. Most data science applications for NCD prevention and control in Africa remain in the exploratory/research phase, thereby limiting real-world clinical or public health utility. Multi-sectoral interventions that promote interdisciplinary capacity building, investment, and knowledge linkages, taking into account indigenous epistemologies, are needed to harness the enormous potential of data science to accelerate the prevention and control of NCDs in Africa.

## Methods

This systematic review was reported using the Preferred Reporting Items for Systematic Review and Meta-Analyses framework^27^. We undertook a systematic review and gap analysis, as registered in PROSPERO (CRD42023406237)^28^ and published elsewhere^29^. The operational definitions of data science and NCDs are detailed in the Supplement. All articles, unique identifiers, and web links are publicly available. Institutional Review Board approval was not required as the study involved analysis of published data.

### Search strategy

Five electronic databases (MEDLINE, EMBASE, Web of Science, African Journal Online, and Cochrane CENTRAL) and one search engine (Google Scholar) were searched from the earliest record through March 2024. Two experienced librarians (A.A.K. and A.G.A.) developed a search plan with the content experts (A.P.O. and M.O.O.). The Librarians conducted a pilot search to assess the sensitivity and specificity of the search strategy, and a full search was then performed. Predefined keywords and MeSH terms were used based on the population, intervention, comparison, and outcomes (PICO) principles^30^. The search strategy, including keywords and MeSH terms, is detailed in the Supplement (Table S1 and Supplementary Data S2). Boolean operators were applied to combine synonymous keywords and MeSH terms as applicable. Truncation was used where relevant. The reference lists of included studies were screened for additional relevant articles. All citations were collated using EndNote^31^. Duplicates were removed using the EndNote automated function and then uploaded to Covidence systematic review software^32^ for screening, full-text review, and data extraction.

### Record screening and selection

**Fig. 1:**
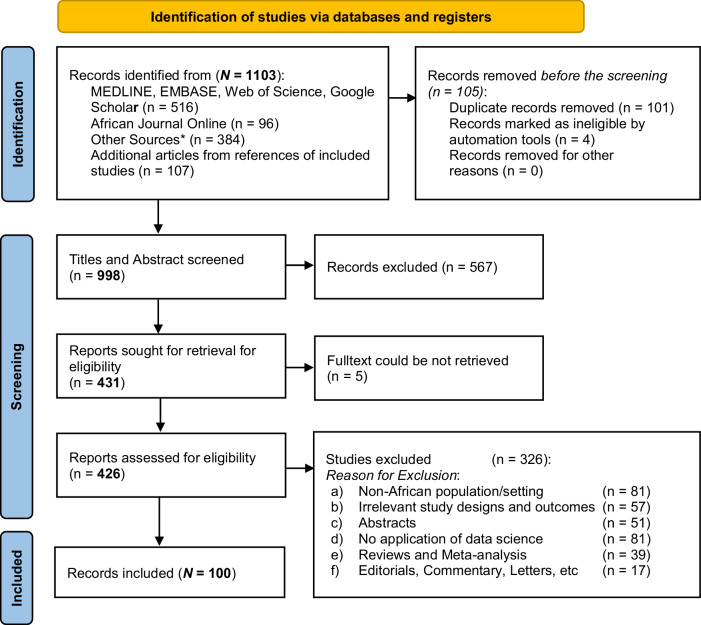
PRISMA flow diagram for identification of articles in this systematic review. *This includes published articles on the subject in the H3Africa Consortium and H3Bionet and published technical reports from organizations such as WHO, Africa CDC, and the African Union.

Three authors (A.P.O., O.J.A., A.G.F.) independently screened all retrieved records, including titles and abstracts, and all inconsistencies were resolved through consensus. Seven authors (A.P.O., O.J.A., A.G.F., E.C., M.I.M., M.T.O., and J.K.) independently reviewed potential articles for full-text assessment and eligibility, and all discrepancies were resolved through a discussion to reach a consensus. Where several articles with the same objective originate from the same dataset, the article with the most comprehensive dataset was selected. The PRISMA flow chart detailing the final selection of articles for the systematic review and gap analysis is presented in Fig. 1.

### Inclusion criteria

Articles were included if they were (1) community- or hospital-based report(s) encompassing biomedical research dataset(s) that uses DSMs for generating, gathering, processing, storing, managing, and analyzing health data, (2) NCDs (including CVDs, cancers, type 2 diabetes and chronic respiratory diseases) as the primary outcome of interest, (3) primarily undertaken among Indigenous Africans, (4) published in a peer-reviewed journal, and (5) with the explicit application of DSMs including omics studies, genome-wide association studies and Mendelian randomization or big data analytics, deep learning methods, machine learning, and artificial intelligence and traditional regression models among others. Included articles may use models for risk prediction, screening or surveillance, diagnosis of NCDs, or prognosis and rely on any combination of temporal, spatial, or biomarker data (e.g., genomic, transcriptomic, proteomic, or radiomic data) for NCDs.

### Exclusion criteria

The following were the basis for excluding articles: (1) articles that applied classical techniques, such as linear, non-linear, autoregressive moving average, logistic, or Poisson regression models, for a test of association with NCD events only; (2) genome-wide association studies restricted to secondary analyses of previously identified genetic variants associated with NCDs in the same dataset; (3) book chapters, conference presentations, reviews, opinions, commentaries and letters to the editor.

### Data extraction

Data was independently extracted using a pre-defined proforma in Covidence by A.P.O., O.J.A., A.G.F., E.C., and M.I.M. Additional rounds of reviews were conducted to verify the quality of the extracted data by A.P.O., O.J.A., A.G.F., and O.O., and all discrepancies were resolved among the review team members. Data extracted included the bibliography (first author’s last name, year of publication, institutional affiliations of first and corresponding authors), study characteristics (country of recruitment, design, and type, among others), DSMs (name, type, sample size, data type, performance metrics, compliance to reporting guidelines, open data sharing information, strength and limitations, among others), NCD outcome of interest (type and the category in the NCD quadrangle) and funding source(s), among others. In consultations with B.A. O.M.A. and MO; OO, OJA, and APO undertook additional data cleaning to clarify terminology discrepancies.

### Methodological and quality assessment

Methodological and quality assessment of potentially eligible articles were independently evaluated by A.P.O., O.J.A., A.G.F., E.C., and M.I.M. using 14-point criteria (including objective, population, eligibility, inclusion criteria, sample size justification, measurement of exposure, and statistical analysis), with a point score for each criterion in keeping with the National Institute of Health study quality assessment tool^33^. All differences were resolved in a discussion. An article was rated “good” (with the least risk of bias) if it attained at least 75% of the overall 14-point score, “medium” (prone to some bias considered insufficient to invalidate its conclusions) with a score between 50%-74% of the overall score, and “poor” (with a significant risk of bias deemed sufficient to invalidate its findings) where the overall score is less than 50%^33^. Studies presenting invalid or inapplicable information on at least nine items on the 14-point scoring criteria were excluded from the assessment.

### Data quality, performance and utility

Several DSMs and definitions were retrieved from the included studies and summarized or classified into five categories: deep learning (DL)^34,35^ only, machine learning (ML)^34–36^, artificial intelligence (AI)^37^, traditional statistics (TS)^38^, and genetic and genome association studies (GGA)^39–42^. The basis for the classification is described fully in the Supplement. Guided by a previous report on medical applications for AI^43^, we rated DSMs or specific models reported in each article under the following themes: data quality (considering the type of data, sample size, and number of DSM reported), model development and validation (training/development only, internal validation and external validation and whether there was any blinding measure, i.e. concealing classifying or outcome data from scientists developing model), and model performance (choice of quantitative performance metric and model with best performance metric where more than one DSM was applied). Furthermore, articles were classified as follows based on the implementation phase and utility for clinical and public health decisions of the DSM: (a) has been recommended for clinical or public health utility, (b) applied in a clinical or public setting (even if it is assisting in decision support), (c) needs clinical and public health validation, and (d) is not ready for clinical and public health application (because it is still at research phase or required standards or quality were not carried out or not reported).

### Determination of funding and collaboration

In assessing the magnitude of intra-African versus trans-continental collaborations on the application of data science for the prevention and control of NCDs, we compared data on the country of origin of data source/participant recruitment and institutional affiliation of the leading (first or corresponding) authors reported in the articles to estimate the proportion of articles having the institutional affiliation of the first author or corresponding as the same as the location of recruitment. Also, we estimated the proportion of articles where the country of data source/recruitment is different from the country of institutional affiliation of the leading author(s) (whether still within Africa or other world regions). Furthermore, we gathered data on reported funding to evaluate the degree of research funding from Africa-based versus international institutions for data science in the prevention and control of NCDs in Africa.

### Evidence gap maps

Evidence gap maps (EGM) were developed using Campbell EGM guidelines^44^ to identify existing research, knowledge and gaps in evidence by the types of DSM applied to the four highlighted NCDs^26^: CVD (including stroke), cancers, type 2 diabetes, and others in Africa within the adapted continuum of care for NCD along the four pillars of the NCD quadrangle: (a) surveillance, determinants, risk prediction, screening/detection, biomarkers, diagnosis; (b) prevention; (c) treatment/drug discovery, prognosis, (d) rehabilitation; and point of care technologies^45,46^. All differences were resolved through discussion among the following authors: APO, OJA, AGF, and MOO. All authors provided input to guide pragmatic recommendations for harnessing data science for NCD prevention and control in Africa, within the context of identified gaps and necessary actions for implementation by relevant stakeholders.

### Data summarization and analysis

The extracted data were reviewed for consistency before being summarised for presentation in tables (in some cases, presenting counts and percentages) and figures as appropriate, using the R Foundation Package for Statistical Computing.

## Results

### Characteristics of included articles

Of the 1103 articles retrieved, 100 (9.1%) were eligible for inclusion (Fig. 1). The list of eligible articles, with the NCD types and DSM, including methodological details, is presented in Supplementary Data S3.

### Data science methods—DSM

To capture the wide range of DSMs reported across several NCD-related studies in the systematic review and gap analysis, we classified the methods into five main categories, including generic artificial intelligence (AI), deep learning (DL) only, other machine learning (ML) only, traditional statistics (TS) and genetic and genome association studies (GGA) not involving the use of AI, ML and DL. Overall, fifty (50) of all included articles reported applying ML^47–96^, thirty-three (33) were on GGA–based methods^90,91,97–127^, thirty-two (32) applied TS models^47,48,55,63,66,71,76,80,81,83,85,86,89,93,96,103,113–116,118,123,128–137^, eighteen (18) on AI^52,61,75,78,91,96,105,107,109,124,128,136,138–143^ and twelve (12) on DL^71,74,79,95,129,138,139,141,142,144–146^.

### NCDs

Cancer-related outcomes were the most studied of the NCDs, 36 (36.0%), followed by CVD, 32 (32.0%) and type 2 diabetes, 20 (20.0%). Other NCD-related outcomes 12 (12.0%) include renal failure, adiposity, cognitive function, and liver function. We noted a significant deficit of articles on chronic respiratory diseases. The specific DSM reported, along with the comprehensive classifications by NCD types, are described in Supplementary Data S3 and Fig. S1.

### Distribution of DSM across regions and NCD subtypes by year of publication in Africa

**Fig. 2:**
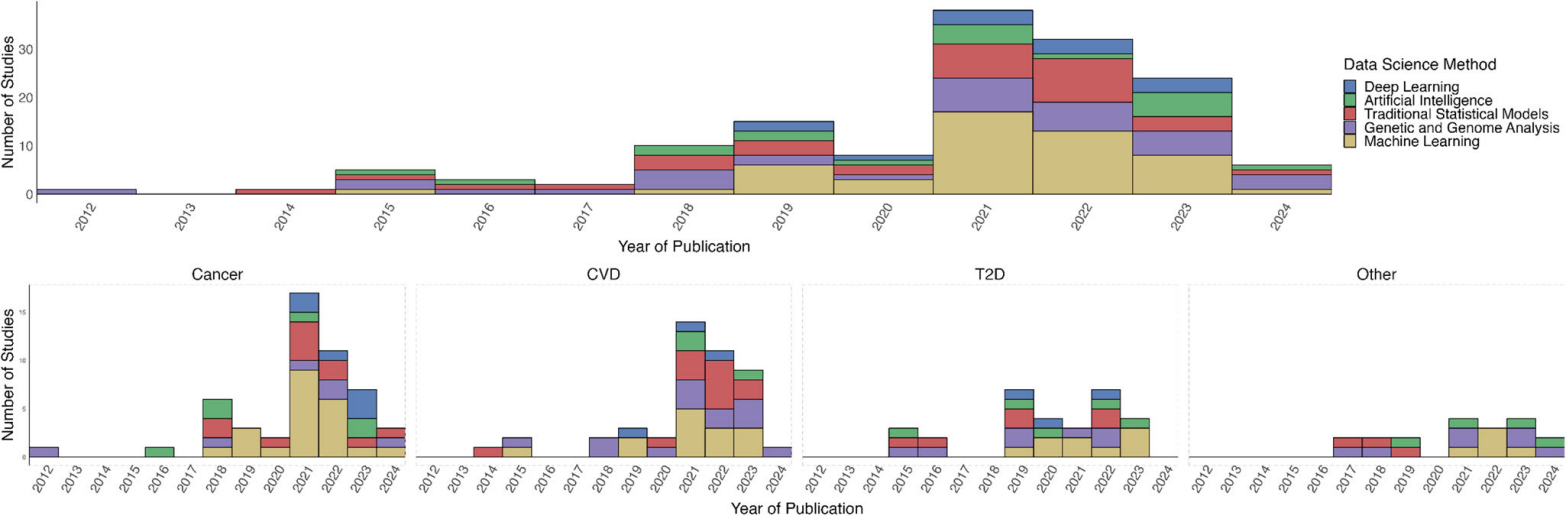
Distribution of studies included by categories of data science method and NCD types across the years of publication.

ML methods in NCD studies represent 50 (50.5%) of all included articles (Fig. 2), though less frequently applied for NCD studies among indigenous Africans before 2018. When the analysis was undertaken by NCD type, ML methods featured prominently in cancer-related outcomes, but TS models were common in CVD-related outcomes. However, we found no consistent pattern in applying DSM for type 2 diabetes and other NCD-related outcomes according to the year of publication.

**Fig. 3:**
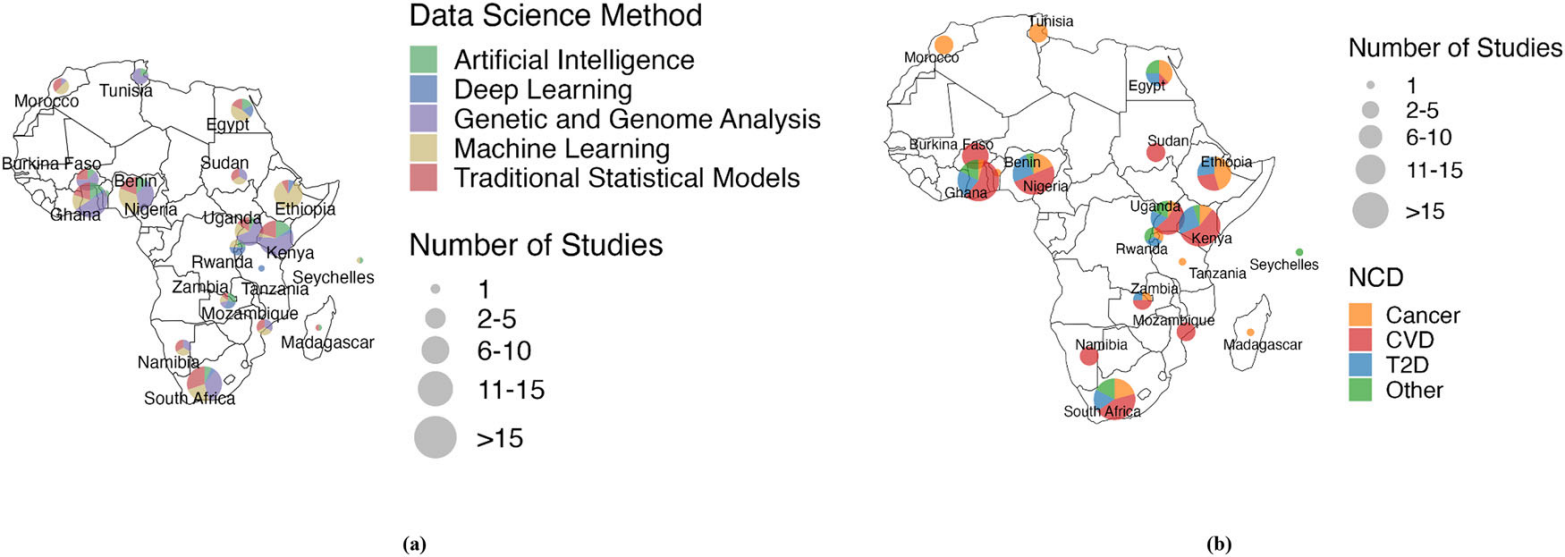
Distribution of included articles by data science methods (**a**) and NCD types (**b**) across African countries in the systematic review.

Still, articles on DSM for NCDs were available in only 19 (out of 54) African countries, with a considerable dearth of reports in Central Africa (Fig. 3a). Moreover, the distribution of studies by DSM and NCD outcomes differed widely by region of Africa. For example, studies from North Africa, especially Morocco and Tunisia, reported multiple DSMs limited to cancer-related outcomes. ML methods were more prevalent among studies from Morocco and Egypt, whereas GGA was widespread among studies from Tunisia (Fig. 3b). However, GGA methods were particularly prominent, especially in relation to CVD-related events in West Africa.

### Methodological assessment

Most DSM 40 (40.0%) were from cross-sectional studies (Fig. S2), and only one (1.0%) was from a randomized trial. Sources of the dataset(s) for DSM by NCD-related studies in Africa include hospital-based 54 (54.0%), community-based 21 (21.0%), and specialized databases including electronic records and employment registries, 5 (5.0%), while the data source was not reported in 5 (5.0%) of all included studies. Although 95.0% of the studies articulated the primary purpose of applying DSM for specific NCD event(s), the underlying inclusion criteria to reduce bias in sample selection were unspecified for most (68.0%) of these studies.

**Fig. 4:**
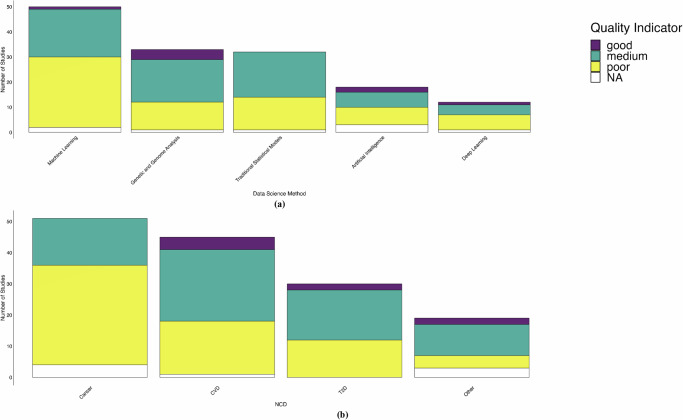
Methodological quality assessment of included studies was carried out using the National Institute of Health Quality Assessment Tool and rated as “poor” (poorly conducted studies with a significant risk of bias deemed sufficient to invalidate the findings likely), “medium” (prone to some bias considered insufficient to likely invalidate the findings) and “good” (well-conducted studies with the least risk of bias) to determine the risk of bias of articles included in the systematic review. The methodological quality assessment report was stratified by (**a**) the data science methods: Machine Learning, Genetic and Genome Association, Traditional Statistics, and Artificial Intelligence and Deep Learning and **b** NCD types: Cancers, CVDs, T2D, and Others, reported in included studies.

Overall, the quality assessment of the epidemiological design of data sources for the DSM revealed that 48 (50.5% of the 95 studies eligible for the methodological evaluation) were of poor quality (Fig. 4a), with ML methods presenting a higher proportion. After stratifying by NCD types (Fig. 4b), more than two-thirds (68%) of studies on cancer-related outcomes were of poor quality.

### Data quality, model performance and utility

Data types were primarily datasets or nomograms of tissues from humans (Supplementary Data S4). Also, 38 (38%) of the included studies had a fair sample size (<1000 samples), and 60 studies applied more than one specific DSM. Almost all studies 98 (98.0%) described the development and training of the DSM, but only 47 (48.0%) and 17 (17.3%) of these studies reported internal or external validation of the DSM, respectively. Notably, no information was provided on any form of blinding in the development or validation of the DSM.

Also, 31.0% of the included studies failed to report any form of validation of model outputs for the DSM. Traditional statistical methods [16 (50.0%)] were the least validated, with the lowest lack of validation observed for DL [2 (18.2%)] and ML [7 (14.0%)]. These findings were similar across NCD types; however, the lack of validation was widespread, particularly for cancer-related outcomes (Fig. S3). Additionally, only 10 (out of 100) included studies reported the open-source availability of the codes to facilitate reproducibility (Fig. S4).

**Fig. 5:**
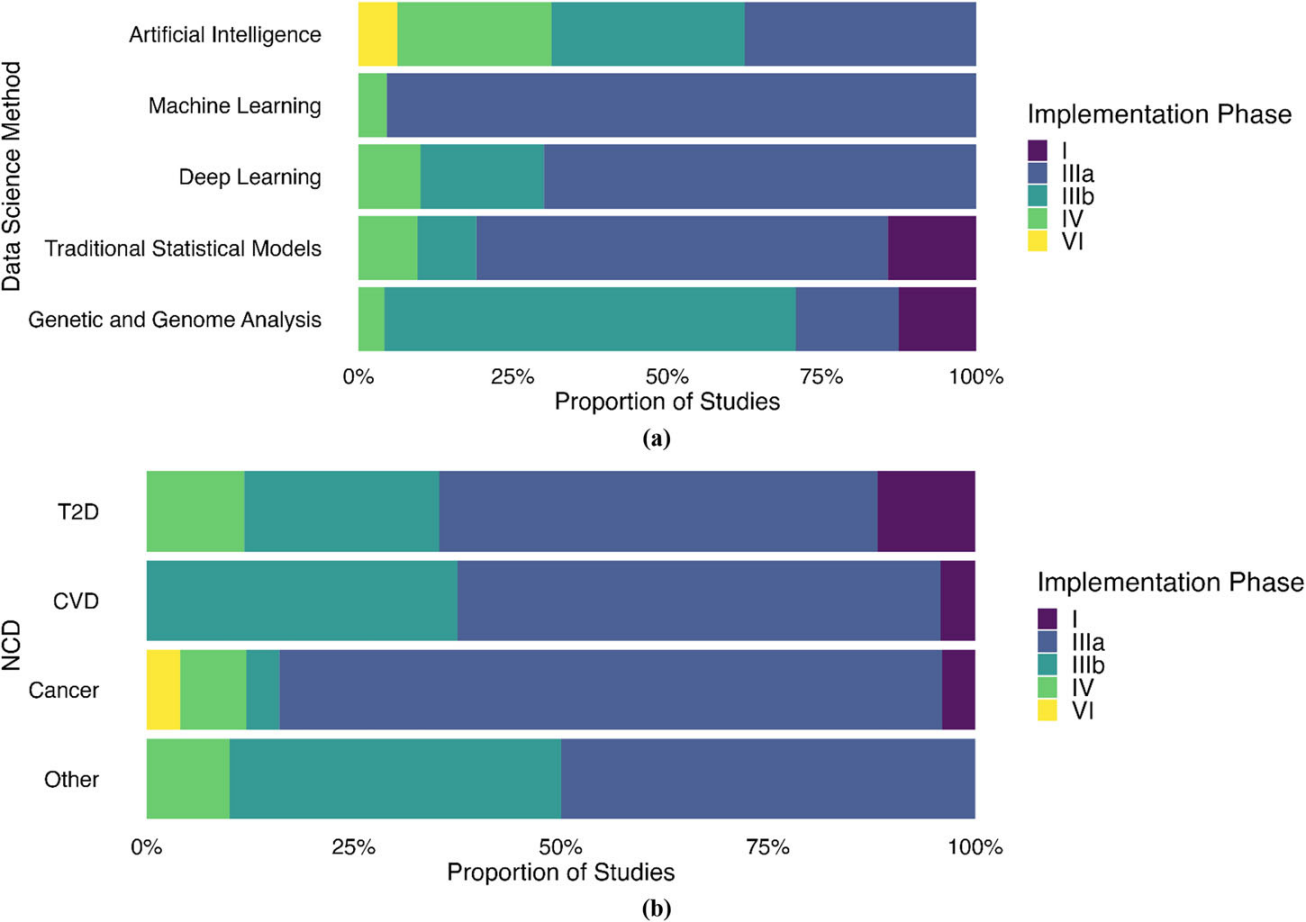
Distribution of included studies by phase of implementation concerning clinical or public health practice for NCD prevention and control according to the data science method and by NCD type. **a** Data science method; **b** NCD type. The phase of implementation phase connotes; I: Data collection and processing; II: Model construction; IIIa: Model validation (Internal only); IIIb: Model validation (Internal and External); IV: Software application development; V: Impact and efficiency analysis; VI; Model implementation in routine Clinical practices/Public Health utility in Health Settings.

Furthermore, 47 (47.0%) studies reported more than one DSM and provided information on whether any form of quality metric(s) was applied in assessing the quality and robustness of models from the DSM (Supplementary Data S4); generally, ML methods showed improved performance in about 38 (80.8%) based on the model performance, especially in comparison with TS methods. Interestingly, the utility (in clinical or public health settings) of most DSM (76 of 100 studies—76.0%) for Africans could not be ascertained, given that most were still in the research phase or required regulatory standards (Fig. 5).

### Funding and collaboration

**Fig. 6:**
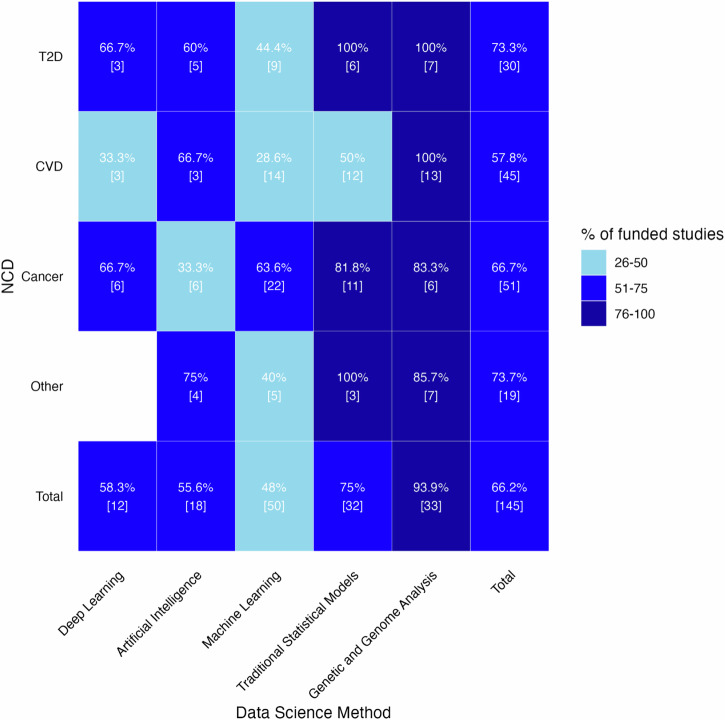
Distribution of funding by NCD type and data science method. All funding information documented in the included studies was collated and stratified by NCD types and data science methods. The white box indicates an empty cell, meaning no studies were recorded in that category (unfunded studies) for the specified NCD type and data science method. The numbers in parentheses indicate the counts for the displayed percentages. The percentages were calculated using the total number of studies in each category as the denominator, reporting the proportion of funded studies only. As a result, percentages for unfunded studies were not included, and the reported percentages do not sum to 100%.

**Fig. 7:**
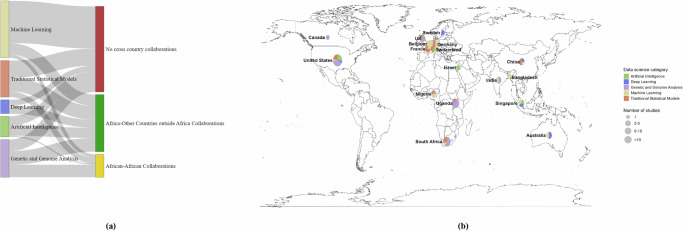
Distribution of collaboration and partnership within and outside Africa on harnessing the benefits of data science for NCD prevention and control in Africa. A Sankey diagram chart describing the nexus of collaborations on how data science has been harnessed for NCD prevention and control in Africa (**a**). The distribution of collaborations on data science for NCD prevention and control in Africa reveals countries where collaborations exist on this phenomenon across Africa and the globe (**b**). In assessing the magnitude of intra-Africa partnerships on the application of data science for NCD prevention and control, we compared data on the country of data source/recruitment and institutional affiliation of the first or corresponding authors reported in the articles to estimate the proportion of articles having the institutional affiliation of the first author or corresponding as the same as the location of recruitment and estimated the proportion of articles where the country of data source/recruitment is different from the country of institutional affiliation of the first or corresponding author (whether still within Africa or other world regions).

Only 66 (92.9%) of the seventy-one (71 out of the total 100) studies that reported funding information received funding (Fig. 6). Of these, 19 (28.7%) were funded by institutions and agencies in Africa. Detailed funding information by DSM and NCD types revealed that GGA-related studies (93.9%) were the most supported, primarily through international funding, while AI-related studies (55.6%) were the least funded. However, ML and AI-related studies were the least funded across NCD types. Furthermore, intra-African collaboration on data science for NCD prevention and control remains the least, with only 15% of the studies presenting a primary institutional affiliation within Africa as the first or corresponding author (Fig. 7).

### Evidence gap mapping (EGM)

**Fig. 8:**
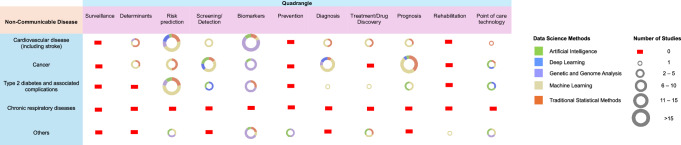
Evidence gap mapping of available studies on the application of data science methods for the prevention and control of non-communicable diseases in Africa. Evidence gap maps (EGM) were developed using Campbell EGM guidelines to identify existing research, knowledge and gaps in evidence by the functionality and types of DSM applied to the four most prominent NCDs: CVD (including stroke), cancers, T2D, and others in Africa within the adapted continuum of care for NCD: surveillance, determinants, risk prediction, screening/detection, biomarkers, prevention, diagnosis, treatment/drug discovery, prognosis, rehabilitation and point of care technologies.

The EGM on data science techniques for NCD prevention and control across the NCD quadrangle in Africa are presented in Fig. 8. Generally, several studies were related to risk prediction (mainly featuring the TS methods) and biomarker determination (primarily based on GGA). However, a shortage of data science research targeted at improving NCD surveillance, identifying determinants, addressing prevention, and treatment/drug discovery and rehabilitation in Africa was evident. Similarly, data science techniques that provide point-of-care technologies for clinical and public health utility are scarce, as very few of these technologies deployed in Africa were designed and developed by, or with contributions from, Africans.

Moreover, TS methods were prominent in exploring determinants, and ML methods featured more in risk prediction for CVD-related events. There was generally a lack of ML, DL and AI methods for cancers, with TS methods prominently featured in cancer risk prediction and prognosis. ML was featured more for risk prediction, diagnosis and treatment of type 2 diabetes-associated complications. Despite these, there was still a scarcity of data science efforts targeted at the critical spectrum of the NCD quadrangle in Africa.

## Discussion

There is evidence of the application of data science, with most efforts evolving, but notable gaps remain in the application of DL and AI techniques for NCD prevention and control in Africa. In addition to a gross lack of funding and limited intra-African partnerships, several methodological issues exist with the current DSM applied to NCD prevention and control in Africa, which complicate the utility of these DSM for clinical/public health decision-making or risk stratification. Our findings present a valuable opportunity to adopt advanced technology-driven healthcare systems by promoting a multi-stakeholder approach that addresses several gaps in harnessing the potential of data science for NCD prevention and control in Africa.

This systematic review and gap analysis reveals the dominance of ML techniques and cancer-related studies, but not without methodological issues that limit generalizability. The application of data science for NCD prevention and control are evolving at a slower pace compared to high-income settings, where they are being used for effective healthcare delivery^147^. Significant gaps exist in the application of data science techniques for type 2 diabetes, which is a leading cause of NCD-related mortality on the continent. Also of note is the shortage of data science for chronic respiratory diseases, which is a major NCD accounting for the morbidity and mortality in Africa. Several factors limit the application of a standard to determine the most viable DSM^148^, but performance metrics generally revealed that ML techniques outperform traditional statistical methods. The broad applications of data science techniques to combat the continent’s high burden of NCDs are underdeveloped due primarily to the lack of quality health data and inadequate funding investments in health infrastructure for harnessing the potential of data science in health outcomes. Besides these, multiple methodological issues, such as poor data generation, quality, and weak model development and validation, also limit the viability of existing data science approaches in NCD prevention and control. Moreover, while guidelines exist for clinical trial reports for interventions involving artificial intelligence^149^ and clinical prediction models^150^, there was a significant lack of compliance to these reporting guidelines. Future studies must address these flaws to minimize potential bias and enhance validity, transparency, and generalizability, thereby promoting transformative clinical decision-making and achieving substantial public health impact for NCD prevention and control in Africa.

Evaluating the quality of research on data science methods for NCD prevention and control is multifaceted, encompassing broad areas of data collection and preprocessing, data splitting into training/validation/test sets, the rationale for model architecture and performance metrics, the robustness and reproducibility of the applied methods, and formal testing of model performance in held-out test data. Validation becomes critical when “black-box” architectures^151^, such as deep convolutional neural models, are used, considering that biologically irrelevant parameters may sometimes drive model fitting, thereby confounding observed performance metrics^152^. Notwithstanding, 31% of studies examined failed to address model validation, and only 17% conducted internal and external validation. Validation becomes challenging in the context of data scarcity, a common problem in Africa^153^. In this setting of limited data, which precludes robust validation, explicit reporting of the inclusion and exclusion criteria can guide further model evaluation and interpretation of performance in external datasets.

Nonetheless, explicit inclusion and exclusion criteria were missing in over 65% of the studies evaluated. This limits the propagation of models, as unvalidated models are unlikely to be applied trans-institutionally. Interdisciplinary discussions among domain experts in data science and statistical methods, as well as healthcare workers who directly handle health and medical data, ideally determine the appropriateness of model architecture. The rationale for choosing specific architectures, such as recurrent neural networks, for natural language processing tasks is based on established evidence of their capacity to handle such data more effectively than other neural network architectures. As a result, explicit reporting of the rationale for model architecture choice may not always be necessary. Also, empirical evidence from testing several models tends to inform the choice of an ideal model. Published research should adhere to reporting standards across critical stages in model development (where applicable) to improve the scientific body of evidence from DSM application for NCD prevention and control in Africa. Reproducibility also eases the external validation of models and model applications at the end-user level. However, only one in ten studies in this systematic review provided open code for reproducing analyses reported in their papers.

The magnitude of these methodological gaps underscores the need for ethical and regulatory oversight to govern the use of data and its application in policy and practice across clinical and public health settings. Researchers should adhere to ethical guidelines that ensure that data collection and usage in data science do not exacerbate existing inequalities in NCD morbidity and mortality^154,155^. The training of AI models typically requires the collection and use of large datasets. Ethical requirements such as patient consent, protection of sensitive data, and privacy, among others, should be implemented^155,156^. Biases in data and algorithms have had a significant social impact on marginalized populations in Western countries. Indeed, there are contextualized guidelines^149,150^, but rarely applied in reporting most studies, for multifactorial reasons such as a lack of awareness, non-compliance and poor ethical oversight. Therefore, the observed methodological fragility in most of these DSMs, which often makes oversimplified causal inferences unsupported by the type and source of data and methodology, is not surprising. These gaps must not be perceived as hindrances, but rather as a golden opportunity to address the likelihood of these data and methodological gaps, thereby promoting potentially unintended deleterious perceptions that could hinder public interest in embracing data science for quality health delivery in NCD prevention and control. Additionally, ethical oversights are necessary, particularly to mitigate the potentially harmful impact of methodological gaps in clinical and public health decisions that affect the lives of patients and populations. It is imperative and imminent for scientists across diverse fields to organize and regulate the data science space in Africa and globally. The regulatory framework should involve designing consensus standard criteria for conducting and reporting data science in observational studies. This framework will ensure quality, transparency, and completeness, thereby earning public trust in harnessing the potential of data science for innovative healthcare delivery.

The cross-cultural applicability of data science in addressing the massive burden of NCDs is quite limited, and our findings suggest that the application of data science has been practically limited to specific within-country populations on the continent. Statistical power and population diversity are crucial for improving the validity and generalizability of findings derived using data science methods. However, a considerable gap in achieving this is the absence of a data harmonization and governance strategy for NCDs, along with relevant phenotypic and genotypic information to improve cross-cultural applicability in using data science across the vast and diverse cultures on the African continent. The absence of rich data infrastructure, particularly across populations in Africa, exists. Where they exist^157,158^, they remain relatively rudimentary^159^. Ethically compliant data governance and harmonization strategies, ensuring the fair representation of diverse indigenous epistemologies (such as Ubuntu Large Language Models^160^) across Africa, are long overdue. This is crucial for promoting health equity in leveraging data science for quality healthcare delivery, including NCD prevention and control, without complex bureaucracies that hinder data access.

We show huge gaps in funding and partnerships. First, funding for data science techniques, especially in harnessing the benefits of ML and AI for NCD-related studies, remains inadequate. Notably, the funding for GGA studies observed in this study is primarily from the National Institutes of Health and Wellcome through the H3Africa Consortium^161^. Nevertheless, the indigenous African genome still represented <5% of the genome-wide association studies catalog worldwide^162^. Besides national efforts in South Africa^163^, Tunisia^164^ and Egypt^165^, international sources primarily provided funding for extensive genomics infrastructure and research in Africa, based on a shared interest in global equity and inclusiveness. Worse still is the deficient of engagement from regional and national governments across Africa in health spending^166^, which hinders the pace for harnessing the potential of data science for NCD prevention and control. Our findings offer a unique opportunity for regional and national governments across Africa to commit to establishing substantial regional- and national-level fiscal resources to provide infrastructure in advancing the gains of data science, including ML with DL and AI, for NCD prevention and control, and other public health challenges in Africa.

Moreover, the minimal intra-Africa partnerships hinder knowledge sharing, method replication across diverse populations, honing local skills, and contextualization and interpretation of information by Africans for Africans in harnessing the benefits of data science for NCD prevention and control in Africa. Under-representation and health inequities in AI applications for healthcare^167^ could be addressed through political, legal, ethical, anthropological and socio-economic frameworks for data science, which will stimulate equitable collaboration and data collection, sharing and application across diverse populations in Africa. Similarly, African-led and domiciled shareable digital platforms that interface with digitised resources (including tools, software, and infrastructures) for training and capacity development, data deposit, integration, sharing, management, computing, and data science skills, among others, are necessary. In addition, concerted efforts promoting intra-African and multidisciplinary collaboration are long overdue to establish sustainable knowledge hubs and foster scientific linkages that facilitate the cross-pollination of ideas, tools, resources, and capacities. This approach will help connect African scientists and address multiple gaps that hinder the reaping of benefits from data science for NCD prevention and control in Africa.

Gaps in evidence exist. The scarcity of data science techniques’ application across various aspects of the NCD quadrangle, including surveillance, identifying determinants, addressing prevention, and treatment/drug discovery and rehabilitation, limits the opportunity to apply its state-of-the-art potential to improve health and address the disproportionate burden of NCDs in Africa. Integrating and deploying data science techniques where gaps exist within the NCD quadrangle would revolutionize NCD prevention and control on the continent^20^. For example, applying data science techniques to NCD surveillance will enable the real-time and longitudinal collection, analysis, and dissemination of information, improving NCD prevention and control^168^, while also bolstering health-system mobilisation for community engagement and public health data science^20^. This will reduce pressure on the already strained, primarily clinically centered healthcare system, particularly within the context of a revolutionized NCD prevention and control approach in Africa^169^. Unlike traditional methods, these systems can facilitate the primordial and primary prevention of NCDs through real-time surveillance and early detection of disease risks, as well as treatment, rehabilitation, and point-of-care solutions^168,170^. Also, there is a considerable deficit of data science methods in chronic respiratory diseases despite its impact on the magnitude of NCD-related disability, morbidity and mortality.

Similarly, the clinical utility and public health relevance of these data science techniques in improving NCD prevention and control remain a global challenge^171^. Thus, additional efforts are necessary by regional and national governments on the continent for strategic interventions to address multiple challenges (such as ethical and data framework, lack of prioritization, and fragmented health system, among others)^172–180^. These interventions will drive the implementation of mechanisms for applying data science techniques across all aspects of the NCD quadrangle, fostering personalized healthcare delivery for NCD preventiona and control in Africa.

We hereby show policy gaps. The African Union data policy framework articulates the critical need for infrastructure(s) for advanced data-driven technologies, such as ML and AI, to promote healthy living^181^. Similarly, the WHO Africa region framework for implementing digital health in Africa identified the inadequacy of digital health infrastructures, expecting that “50% of Member States have developed and costed their national digital health architecture” by 2023^182^. This goal, among others, is far from being achieved due to several issues primarily attributable to a lack of specific guidelines and roadmaps to harness these advanced data science methods for disease prevention and control in Africa, as our findings revealed the gross underutilization and critical deficit in the clinical or public utility of most of the data science methods, especially DL and AI targeted for NCD prevention and control.

Our study has some limitations. Studying data science applications across the myriad of NCDs in a single review could derail the intention of this paper. This was avoided by focusing principally on four main NCDs contributing to the most significant burden of diseases within the 4 × 4 framework of health research and programming in the African region. Given that “data science” is a broad registers in the literature, some studies might have been missed during our literature search. However, the use of unique registers and interrelated search terms across five electronic databases and a search engine minimized the likelihood of this. Additionally, some studies (especially those from non-English-based settings, indexed with non-English keywords and registers) might have been missed during the literature search, despite efforts to ensure a comprehensive literature search across Africa, regardless of the language of publication. However, no article was excluded as a result of the language of publication other than English, even though articles found not to be reported in other languages were excluded based on non-relevance during the screening of titles and abstracts. Classifying DSM was complex in light of the interrelated methodologies or terms reported in the included studies, such as data science; however, this was overcome by stipulating operational definitions for classifying studies based on the DSM reported. GGA is a complex general term encompassing most genomic and omics-related processes with multiple data-related components, as defined for this study. Also, exploring GGA studies revealed the shortage of epigenome-wide association and microbiome studies conducted among Africans.

**Fig. 9:**
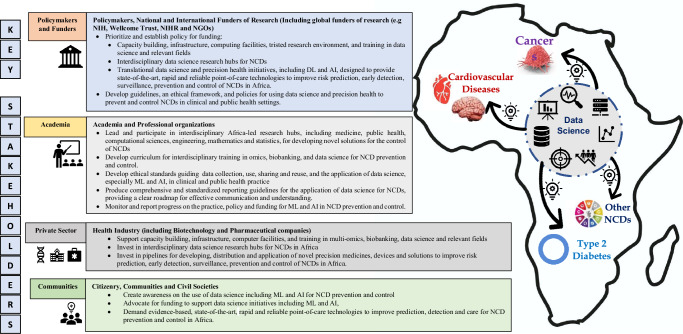
Next Steps for Harnessing Data Science for NCD Prevention and Control in Africa using a Quadruple Helix Approach.

We recommend as follows. Multi-sectoral interventions promoting interdisciplinary capacity building and investment in research, leveraging local epistemologies for culturally relevant interventions, are urgently needed to harness the enormous potential of data science to accelerate NCD prevention and control in Africa (Supplementary Data S5 and Fig. 9). Policymakers and research funders should prioritize national and regional plans, as well as regional hubs, for translational data science and precision health initiatives. This should include DL and AI, designed to provide state-of-the-art, rapid and reliable point-of-care technologies to improve risk prediction, early detection, surveillance, prevention, treatment and care for NCDs. This should cater for capacity building, infrastructure, computing facilities, research environments, and training in data science and relevant fields. Researchers and professional organizations should establish interdisciplinary research, training, and mentorship networks and ecosystems that encompass medicine, public health, computational sciences, engineering, mathematics, and statistics to develop novel solutions for preventing and controlling NCDs. Industry partners, including biotechnology companies, should prioritise investment in intra-African, multidisciplinary, and interdisciplinary data science research hubs and pipelines for developing, distributing, and applying novel precision medicines, devices, and solutions to improve risk prediction, early detection, surveillance, prevention, diagnosis, treatment, care, and prognosis of NCDs. The general population should be sensitized to participate in relevant research and advocate for harnessing data science to improve the prevention and control of NCDs in Africa. Additionally, dissemination of policy briefs, organization of multi-stakeholder workshops, focus group discussions, meetings, and policy fora, among others itemizing the recommendations of this work for the attention of relevant policymakers, national and regional health authorities and funding agencies in Africa is necessary for making an economic case for investment in addressing infrastructure deficits, funding inadequacies, and capacity-building, among other issues outlined is indispensable.

## Conclusion

This systematic review evaluated the current state of DSM for NCD prevention and control in Africa and found that most data science innovations for shaping health outcomes in NCD prevention and control are still primarily in the research phase and have little clinical or public health utility for a direct impact on the African population. This is likely due to several methodological, infrastructural, funding, capacity and policy gaps that multilateral stakeholders (including researchers, funding bodies, national, regional and global organizations, and the general African populace) must address to leverage the huge potential of data science for NCD prevention and control in Africa. Relevant leading authorities for coordinating for global public health, including WHO, African Union and health ministries in Africa and beyond, can lead by investing in governance, capacity building, ethical innovation, and the equitable deployment of data science, including ML and AI, as transformative tools for NCD prevention and control, especially in anticipating and addressing emerging threats by providing uniform guidelines and framework to organize and standardize data-driven NCD surveillance, forecasting, and intervention tools for the centralization of NCD-related datasets across member states in Africa, developing African-wide ML and AI protocols, taking into account ethics and health equity principles, without compromising sensitive health data and support training and capacity building for health personnel and data scientist and facilitate collaboration for knowledge and technology transfer.

## Supplementary information

Supplementary Information

Description of Additional Supplementary files

Supplementary Data 1

Supplementary Data S2

Supplementary Data S3

Supplementary Data S4

Supplementary Data S5

## Data Availability

Data sharing is not applicable to this article, as no datasets were generated or analyzed during the current study. However, all source data can be accessed in the Supplementary Information. The source data for Figs. 2, 3a, 3b, 4a, 4b, 5a, 5b, 6, 7a, 7b, and 8 are in Supplementary Data 1.
