# Supplementary material for: Harnessing data science to control non-communicable diseases in Africa: a systematic review and gap analysis
Source: Commun Med (Lond). 2026 Jul 16;6:397. doi: 10.1038/s43856-025-01272-0 (PMC13377181; doi:10.1038/s43856-025-01272-0)
Supplement: Supplementary file 1 — Supplementary Information [file 43856_2025_1272_MOESM1_ESM.pdf]

Supplementary Information

Harnessing Data Science to Control Non-Communicable Diseases in Africa: A Systematic Review and Gap Analysis

Supplementary Methods

Operational definitions of terms

Data Science

According to the NIH, data science is "the interdisciplinary field of inquiry in which quantitative and analytical approaches, processes, and systems are used to generate knowledge or insights from large and complex data sets"[1]. Data Science involves the generation and analysis of substantial quantities of biomedical research data, including numerous quantitative and qualitative datasets emanating from fundamental research using model organisms (e.g., mice, fruit flies, zebrafish), clinical studies (e.g., medical images), epidemiological studies (routinely collected health data) and genetic studies (i.e., large scale genomic data). Given the absence of large and complex datasets from indigenous African populations, the operational application of "large and complex data sets" for including studies on data science was omitted in this systematic review.

Data Science Methods

Data science methods (DSM) reported in eligible studies were classified into five categories, each with distinct definitions.

Artificial Intelligence (AI): Included studies were classified as AI, having reported using any form of technologies, devices, and types of equipment designed to imitate various complex skills[2,3] for NCD control and prevention within the NCD quadrangle: surveillance, determinants, risk prediction, detection/biomarkers, prevention, diagnosis, treatment/drug discovery, prognosis and rehabilitation[4,5]. In addition, it implies any study that reported the application of any form of autonomous system(s) or device(s) independently applied to any task within the NCD quadrangle (with minimal inputs/contributions from humans) for NCD control and prevention among indigenous Africans.

Machine Learning (ML): DSM reported in included studies were classified as ML, having reported applying any form of method drawing from logical, empirical or control theory (which includes, but is not limited to, statistics, computer science, and clinical theory, among others)[2,6,7] to improve any task in the retinue of event(s) within the NCD quadrangle (including surveillance, determinants, risk prediction, detection/biomarkers, prevention, diagnosis, treatment/drug discovery, prognosis and rehabilitation[4,5]) for NCD control and prevention using epidemiological dataset among indigenous Africans. The ML method may be supervised, unsupervised learning or reinforcement, deployed for prediction or classification[8] of any NCD-related traits or event(s) among indigenous Africans.

Deep Learning (DL): DSM reported in included studies were classified as DL, having noted it applied traditional statistical or mathematical learning theory or analysis/process to estimate a probability decision or distribution organize information using epidemiological datasets to develop a programme or algorithm[2,6] to manage any task in the course of event(s) within the NCD quadrangle (including surveillance, determinants, risk prediction, detection/biomarkers,

prevention, diagnosis, treatment/drug discovery, prognosis and rehabilitation[4,5]) for NCD control and prevention among Indigenous Africans.

Traditional Statistical (TS): DSM reported in included studies were classified as TS upon reporting the application of any form of method drawing from logical, empirical or control theory (which comprises perceptron, applied, empirical, algorithmic, or theoretical analysis/model, among others)[9] to reveal causal associations with any NCD-related traits or event(s) among indigenous Africans. Such association(s) included any event(s) within the NCD quadrangle (including surveillance, determinants, risk prediction, detection/biomarkers, prevention, diagnosis, treatment/drug discovery, prognosis and rehabilitation[4,5]) for NCD control and prevention using epidemiological datasets among indigenous Africans. These methods include regression, classification, linear model selection, survival analyses or tree-based methods.

Genetic and Genome Association studies (GGA): The DSM reported in an included study was classified as GGA if the research approach adopted surveying genomic (set of DNA sequence or instructions) variants and liabilities, including sequences, maps, chromosomes, assemblies or annotations statistically linked with a specific NCD or related traits with the primary interest of extending frontiers of information on the viability of genetic variants in the prevention and control of NCDs using epidemiological dataset(s). This includes genome sequencing, genome-wide association studies[10], Mendelian randomization studies[11], or polygenic risk scores[12], among others, methodologically designed using computational methods[13] to provide a myriad of robust evidence on the heritability of single or multiple genetic variants or causal links with NCD or related traits among indigenous Africans.

Non-communicable diseases (NCDs)

NCDs represent a spectrum of diseases[14] and are a leading cause of mortality and disability, accounting for a more significant proportion of years of life lost from all disease burdens globally[15]. Similarly, NCD accounts for 67% of total disability-adjusted life-years in Africa between 1990 and 2017[16], and in addition to infectious diseases, imposes a considerable burden of preventable illness on the health system[17]. Specifically, this systematic review primarily focused (but was not limited to) the four most prominent NCDs, including cardiovascular diseases (CVDs), cancers, diabetes, and chronic respiratory diseases with shared risk factors based on the 4 ×4 framework of NCDs public health research and programming by the WHO[18] which was also reflective of the burden of NCDs with the highest DALYs burden in Africa[16]. For example, CVDs are the most prominent of the five well-established NCDs (including stroke, diabetes mellitus, mental health and neurological disorders)[15,19-22], accounting for 5.5% of all CVD-related deaths worldwide and 11.3% of all deaths in Africa[23,24].

NCD Quadrangle Definitions

Surveillance

An included study is classified under surveillance, where the DSM was deployed for monitoring, identifying early signs, and analyzing data on the incidence, prevalence, morbidity, survival, and mortality of NCDs in an African population[25]. It could also involve the application of the DSM for the systematic collection, interpretation, and dissemination of data to target and monitor interventions for NCD surveillance.

Determinants

Where the DSM reported in included studies was planned to identify characteristic(s) that influence the pathophysiology or manifestation of an NCD, they are classified as determinants. Such studies involve identifying risk or causal factors leading to the onset of NCD[26]. The determinants could be multifactorial, ranging from demographic, lifestyle, socioeconomic, behavioural, and environmental factors, among others, that are vital in individual or population-level primordial NCD prevention[27].

Risk Prediction

Studies classified as risk prediction included those that applied the DSM to estimate the future probability of developing one or more NCD event(s). It includes but is not limited to using and identifying clinically valid or empirically viable characteristics (for example, demographic, lifestyle, and biomarkers, among others) associated with the risk of NCD onset using contextually specific data-driven models to estimate future risk of developing NCD or NCD-related outcomes, and to distinguish individuals at risk of NCD[27].

Screening and Detection

Screening and detection apply to studies using DSM to identify or assess for NCDs, especially where no symptoms were observed[28]. The screening methods do not necessarily diagnose NCD in any way; however, such studies have utilised the DSM to provide vital information required for diagnosis and treatment[29].

Biomarkers

Biomarkers apply to studies employing DSM to measure characteristic(s) evaluated as an indicator of normal biological or pathogenic processes or responses to an exposure or intervention[30]. It could be diagnostic, prognostic or predictive biomarkers determination for valuable insights into NCD susceptibility, progression, and treatment response, for drug development and personalized medicine[30,31].

Prevention

Prevention includes studies employing DSM to eradicate, eliminate or reduce the likelihood or impact of NCDs. It could consist of primordial, primary, secondary or tertiary prevention contingent upon the application of the DSM to prevent NCD among Africans[26].

Diagnosis

Diagnosis includes studies using DSM to ascertain the nature and magnitude of NCDs and distinguish them from other possible conditions[32].

Treatment and Drug Discovery

Treatment and Drug Discovery encompasses studies utilizing DSM for the care, management, and control of NCD and its associated complications, aiming to cure, ameliorate, or slow the progression of the disease in most cases after diagnosis. It could include the deployment of DSM to promote efficiency and improve care administration, management, and control of NCD and its associated complications, as well as to address physical symptoms and, in some cases, slow down the underlying pathophysiological processes of NCD[33].

Prognosis

Prognostic-based NCD studies employed DSM to predict NCD progression post-diagnosis or post-treatment, including monitoring or predicting alterations in signs and symptoms to determine the course of improvement or deterioration, taking into account multiple factors to inform appropriate management or treatment for recovery[34].

Rehabilitation

Rehabilitation-related studies are those that apply DSM to restore the loss of any form of functioning, which could be mental, physical, or psychological, among others, lost to NCD. Additionally, it included studies employing DSM to understand multidisciplinary approaches for preventing hospitalization, reducing hospital length of stay, and improving home independence and care[35,36].

Point-of-Care Technologies

Point-of-care technologies include studies employing DSM to offer out-of-laboratory/clinic, state-of-the-art, quick, rapid and reliable diagnosis for NCDs to improve care and treatment, thereby reducing the time and resources required to help patients, allowing for more collaborative and informed care, and providing patients with accessible and affordable diagnostic support that allows them to receive fast and suitable treatment and provide high-quality assessment or measurement optimized for diverse settings without compromising service or treatment quality[37-39].

Africa

The United Nations defines the African region as comprising North, Central, East, South and West African countries[40]. All studies reporting the application of data science in NCD prevention and control among indigenous African populations within the territorial boundaries of Africa were considered in this systematic review.

Table S1: Keywords, MESH terms applied in the preliminary search strategy in the scientific databases

S/N	Database	Search Strategy/Terms/Query[1]
1	MEDLINE (PubMed)	('Data science' OR 'research data management' OR RDM OR 'data curation' OR 'data manipulation' OR 'big data' OR 'open data' OR 'artificial intelligence' OR 'data modelling' OR 'machine learning') AND (Cardiovascular diseases OR NCDs OR 'non-communicable diseases' OR 'cardiovascular diseases' OR 'cerebrovascular disease' OR 'ischaemic heart disease' OR 'coronary artery disease' OR 'peripheral vascular disease' OR 'congenital heart disease' OR 'rheumatic heart disease' OR 'cardiomyopathies' OR 'cardiac arrhythmias' OR Cancer* OR 'Diabetes' OR 'heart diseases' OR 'hypertension' OR 'mental health ailments diseases' OR 'stroke*' OR 'hypertension' OR 'hypercholesterolemia' OR 'hyperlipidaemia') AND (Africa* OR Algeria OR Angola OR Benin OR Botswana OR 'Burkina Faso' OR Burundi OR Cameroon OR 'Canary Islands' OR 'Cape Verde' OR 'Central African Republic' OR Chad OR Comoros OR Congo OR 'Democratic Republic of Congo' OR Djibouti OR Egypt OR 'Equatorial Guinea' OR Eritrea OR Ethiopia OR Gabon OR Gambia OR Ghana OR Guinea OR 'Guinea Bissau' OR 'Ivory Coast' OR 'Cote d*' OR Jamahiriya OR Jamahiriya OR Kenya OR Lesotho OR Liberia OR Libya OR Libya OR Madagascar OR Malawi OR Mali OR Mauritania OR Mauritius OR Mayotte OR Morocco OR Mozambique OR Mozambique OR Namibia OR Niger OR Nigeria OR Principe OR Reunion OR Rwanda OR 'Sao Tome' OR Senegal OR Seychelles OR 'Sierra Leone' OR Somalia OR 'South Africa' OR 'St Helena' OR Sudan OR Swaziland OR Tanzania OR Togo OR Tunisia OR Uganda OR 'Western Sahara' OR Zaire OR Zambia OR Zimbabwe OR 'Central Africa' OR 'Central African' OR 'West Africa' OR 'West African' OR 'Western Africa' OR 'Western African' OR 'East Africa' OR 'East African' OR 'Eastern Africa' OR 'Eastern African' OR 'North Africa' OR 'North African' OR 'Northern Africa' OR 'Northern African' OR 'South African' OR 'Southern Africa' OR 'Southern African' OR 'sub Saharan Africa' OR 'sub Saharan African' OR 'sub-Saharan Africa' OR 'sub-Saharan African') NOT ('guinea pig' OR 'guinea pigs' OR 'aspergillums Niger')
2	EMBASE	('Data science' OR 'research data management' OR RDM OR 'data curation' OR 'data manipulation' OR 'big data' OR 'open data' OR 'artificial intelligence' OR 'data modelling' OR 'machine learning') AND (Cardiovascular diseases OR NCDs OR 'non-communicable diseases' OR 'cardiovascular diseases' OR 'cerebrovascular disease' OR 'ischaemic heart disease' OR 'coronary artery disease' OR 'peripheral vascular disease' OR 'congenital heart disease' OR 'rheumatic heart disease' OR 'cardiomyopathies' OR 'cardiac arrhythmias' OR Cancer* OR 'Diabetes' OR 'heart diseases' OR 'hypertension' OR 'mental health ailments diseases' OR 'stroke*' OR 'hypertension' OR 'hypercholesterolemia' OR 'hyperlipidaemia') AND (Africa* OR Algeria OR Angola OR Benin OR Botswana OR 'Burkina Faso' OR Burundi OR Cameroon OR 'Canary Islands' OR 'Cape Verde' OR 'Central African Republic' OR Chad OR Comoros OR Congo OR 'Democratic Republic of Congo' OR Djibouti OR Egypt OR 'Equatorial Guinea' OR Eritrea OR Ethiopia OR Gabon OR Gambia OR Ghana OR Guinea OR 'Guinea Bissau' OR 'Ivory Coast' OR 'Cote d*' OR Jamahiriya OR Jamahiriya OR Kenya OR Lesotho OR Liberia OR Libya OR Libya OR Madagascar OR Malawi OR Mali OR Mauritania OR Mauritius OR Mayotte OR Morocco OR Mozambique OR Mozambique OR Namibia OR Niger OR Nigeria OR Principe OR Reunion OR Rwanda OR 'Sao Tome' OR Senegal OR Seychelles OR 'Sierra Leone' OR Somalia OR 'South Africa' OR 'St Helena' OR Sudan OR Swaziland OR Tanzania OR Togo OR Tunisia OR Uganda OR 'Western Sahara' OR Zaire OR Zambia OR Zimbabwe OR 'Central Africa' OR 'Central African' OR 'West Africa' OR 'West African' OR 'Western Africa' OR 'Western African' OR 'East Africa' OR 'East African' OR 'Eastern Africa' OR 'Eastern African' OR 'North Africa' OR 'North African' OR 'Northern Africa' OR 'Northern African' OR 'South African' OR 'Southern Africa' OR 'Southern African' OR 'sub Saharan Africa' OR 'sub Saharan African' OR 'sub-Saharan Africa' OR 'sub-Saharan African') NOT ('guinea pig' OR 'guinea pigs' OR 'aspergillums Niger')
3	Web of Science	TS=('Data science' OR 'research data management' OR RDM OR 'data curation' OR 'data manipulation' OR 'big data' OR 'open data' OR 'artificial intelligence' OR 'data modelling' OR 'machine learning') AND TS=('Cardiovascular diseases' OR NCDs OR 'non-communicable diseases' OR 'cardiovascular diseases' OR 'cerebrovascular disease' OR 'ischaemic heart disease' OR 'coronary artery disease' OR 'peripheral vascular disease' OR 'congenital heart disease' OR 'rheumatic heart disease' OR 'cardiomyopathies' OR 'cardiac arrhythmias' OR Cancer* OR 'Diabetes' OR 'heart diseases' OR 'hypertension' OR 'mental health ailments diseases' OR 'stroke*' OR 'hypertension' OR 'hypercholesterolemia' OR 'hyperlipidaemia') AND TS=((Africa* OR Algeria OR Angola OR Benin OR Botswana OR 'Burkina Faso' OR Burundi OR Cameroon OR 'Canary Islands' OR 'Cape Verde' OR 'Central African Republic' OR Chad OR Comoros OR Congo OR 'Democratic Republic of Congo' OR Djibouti OR Egypt OR 'Equatorial Guinea' OR Eritrea OR Ethiopia OR Gabon OR Gambia OR Ghana OR Guinea OR 'Guinea Bissau' OR 'Ivory Coast' OR 'Cote d*' OR Jamahiriya OR Jamahiriya OR Kenya OR Lesotho OR Liberia OR Libya OR Libya OR Madagascar OR Malawi OR Mali OR

		Mauritania OR Mauritius OR Mayotte OR Morocco OR Mozambique OR Mozambique OR Namibia OR Niger OR Nigeria OR Principe OR Reunion OR Rwanda OR 'Sao Tome' OR Senegal OR Seychelles OR 'Sierra Leone' OR Somalia OR 'South Africa' OR 'St Helena' OR Sudan OR Swaziland OR Tanzania OR Togo OR Tunisia OR Uganda OR 'Western Sahara' OR Zaire OR Zambia OR Zimbabwe OR 'Central Africa' OR 'Central African' OR 'West Africa' OR 'West African' OR 'Western Africa' OR 'Western African' OR 'East Africa' OR 'East African' OR 'Eastern Africa' OR 'Eastern African' OR 'North Africa' OR 'North African' OR 'Northern Africa' OR 'Northern African' OR 'South African' OR 'Southern Africa' OR 'Southern African' OR 'sub Saharan Africa' OR 'sub Saharan African' OR 'sub-Saharan Africa' OR 'sub-Saharan African') NOT ('guinea pig' OR 'guinea pigs' OR 'aspergillums Niger'))
4	Google Scholar	('Data science' OR 'research data management' OR RDM OR 'data curation' OR 'data manipulation' OR 'big data' OR 'open data' OR 'artificial intelligence' OR 'data modelling' OR 'machine learning') AND (Cardiovascular diseases OR NCDs OR 'non-communicable diseases' OR 'cardiovascular diseases' OR 'cerebrovascular disease' OR 'ischaemic heart disease' OR 'coronary artery disease' OR 'peripheral vascular disease' OR 'congenital heart disease' OR 'rheumatic heart disease' OR 'cardiomyopathies' OR 'cardiac arrhythmias' OR Cancer* OR 'Diabetes' OR 'heart diseases' OR 'hypertension' OR 'mental health ailments diseases' OR 'stroke*' OR 'hypertension' OR 'hypercholesterolemia' OR 'hyperlipidaemia') AND (Africa* OR Algeria OR Angola OR Benin OR Botswana OR 'Burkina Faso' OR Burundi OR Cameroon OR 'Canary Islands' OR 'Cape Verde' OR 'Central African Republic' OR Chad OR Comoros OR Congo OR 'Democratic Republic of Congo' OR Djibouti OR Egypt OR 'Equatorial Guinea' OR Eritrea OR Ethiopia OR Gabon OR Gambia OR Ghana OR Guinea OR 'Guinea Bissau' OR 'Ivory Coast' OR 'Cote d*' OR Jamahiriya OR Jamahiriya OR Kenya OR Lesotho OR Liberia OR Libya OR Libya OR Madagascar OR Malawi OR Mali OR Mauritania OR Mauritius OR Mayotte OR Morocco OR Mozambique OR Mozambique OR Namibia OR Niger OR Nigeria OR Principe OR Reunion OR Rwanda OR 'Sao Tome' OR Senegal OR Seychelles OR 'Sierra Leone' OR Somalia OR 'South Africa' OR 'St Helena' OR Sudan OR Swaziland OR Tanzania OR Togo OR Tunisia OR Uganda OR 'Western Sahara' OR Zaire OR Zambia OR Zimbabwe OR 'Central Africa' OR 'Central African' OR 'West Africa' OR 'West African' OR 'Western Africa' OR 'Western African' OR 'East Africa' OR 'East African' OR 'Eastern Africa' OR 'Eastern African' OR 'North Africa' OR 'North African' OR 'Northern Africa' OR 'Northern African' OR 'South African' OR 'Southern Africa' OR 'Southern African' OR 'sub Saharan Africa' OR 'sub Saharan African' OR 'sub-Saharan Africa' OR 'sub-Saharan African') NOT ('guinea pig' OR 'guinea pigs' OR 'aspergillums Niger')

Supplementary Results

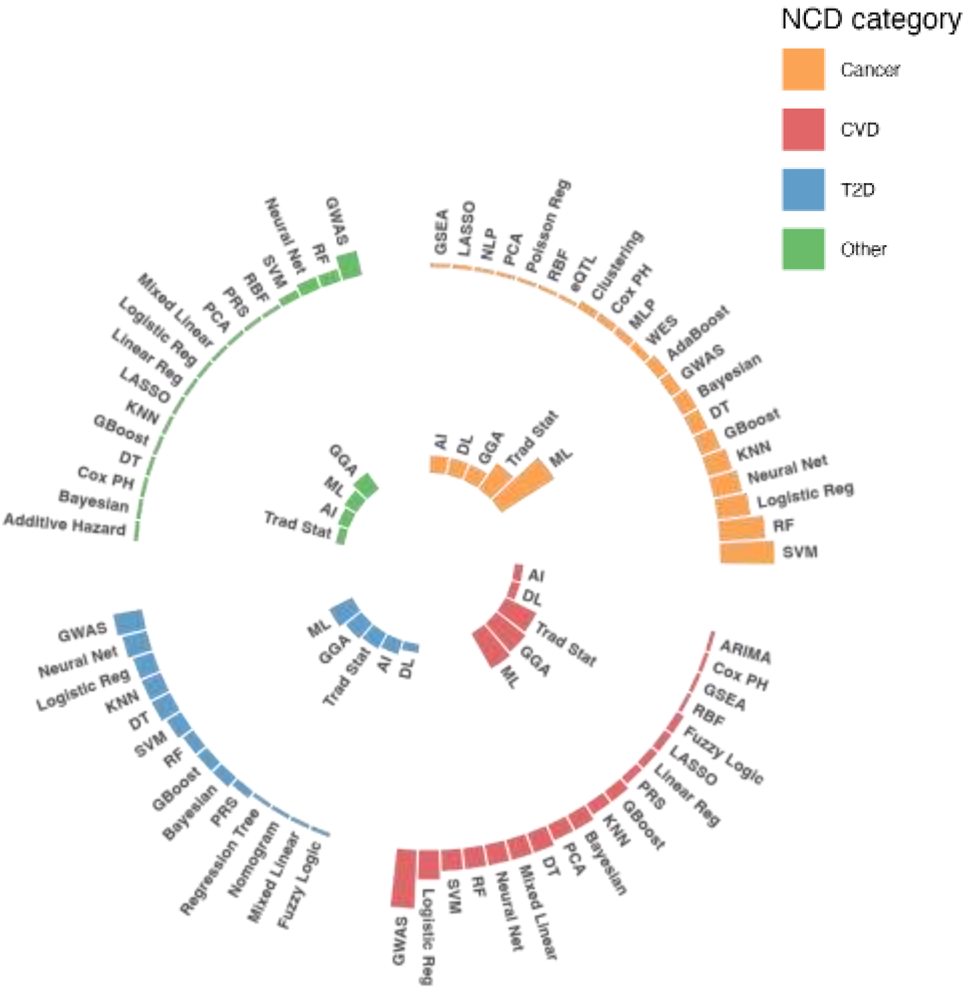

Figure S1: Distribution of methods reported in included studies by the NCD categories within the broad classification of data science methods

AdaBoost: adaptive boosting model, ARIMA: autoregressive moving average model, Bayesian: Bayesian analysis, Clustering: cluster analysis, Cox PH: Cox proportional hazard, CVD: cardiovascular diseases, DT: decision tree, eQTL: expression quantitative trait loci analysis, GBoost: gradient boosting, GSEA: gene set enrichment analysis, GWAS: genome-wide association studies, KNN: K-nearest neighbour, LASSO: least absolute shrinkage and selection operator regression, Linear Reg: linear regression, Logistic Reg: logistic regression, Mixed Linear: mixed linear model, MLP: multilayer perceptron, Neural Net: neural network model, NLP: natural language processing, PCA: principal component analysis, Poisson Regression: generalized linear model form of regression analysis, PRS: polygenic risk score, RBF: radial basis function, RF: random forest, SVM: support vector machine, T2D; type 2 diabetes, WES: whole exome sequencing,

Others include NCD-related outcomes such as adiposity, cognitive function, renal failure, and liver function.

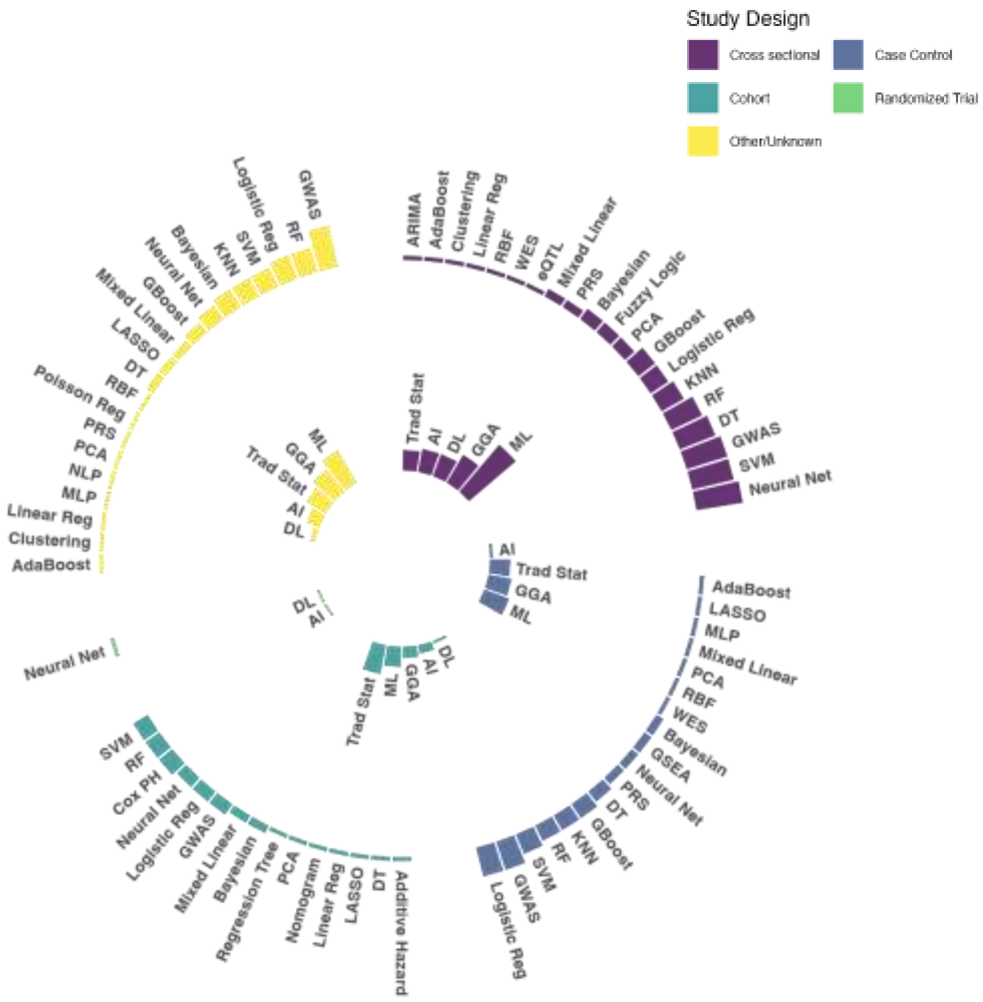

Figure S2: Distribution of methods reported in included studies by the study design within the broad classification of data science methods.

AdaBoost: adaptive boosting model, ARIMA: autoregressive moving average model, Bayesian: Bayesian analysis, Clustering: cluster analysis, Cox PH: Cox proportional hazard, CVD: cardiovascular diseases, DT: decision tree, eQTL: expression quantitative trait loci analysis, GBoost: gradient boosting, GSEA: gene set enrichment analysis, GWAS: genome-wide association studies, KNN: K-nearest neighbour, LASSO: least absolute shrinkage and selection operator regression, Linear Reg: linear regression, Logistic Reg: logistic regression, Mixed Linear: mixed linear model, MLP: multilayer perceptron, Neural Net: neural network model, NLP: natural language processing, PCA: principal component analysis, Poisson Regression: generalized linear model form of regression analysis, PRS: polygenic risk score, RBF: radial basis function, RF: random forest, SVM: support vector machine, T2D; type 2 diabetes, WES: whole exome sequencing.

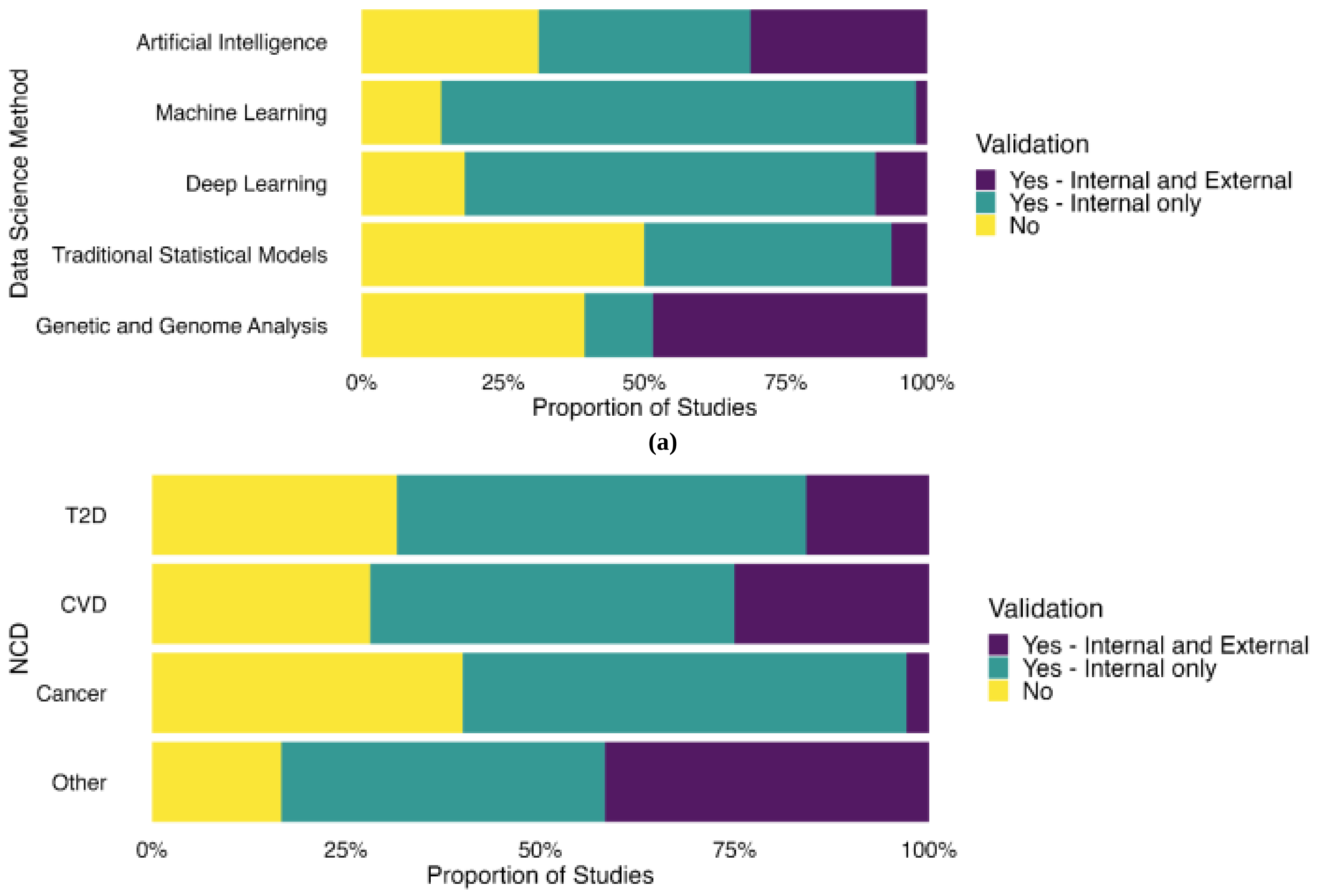

(a)

(b)

Figure S3: Distribution of Validation by Data Science Methods (a) and NCD (b) categories across countries in Africa

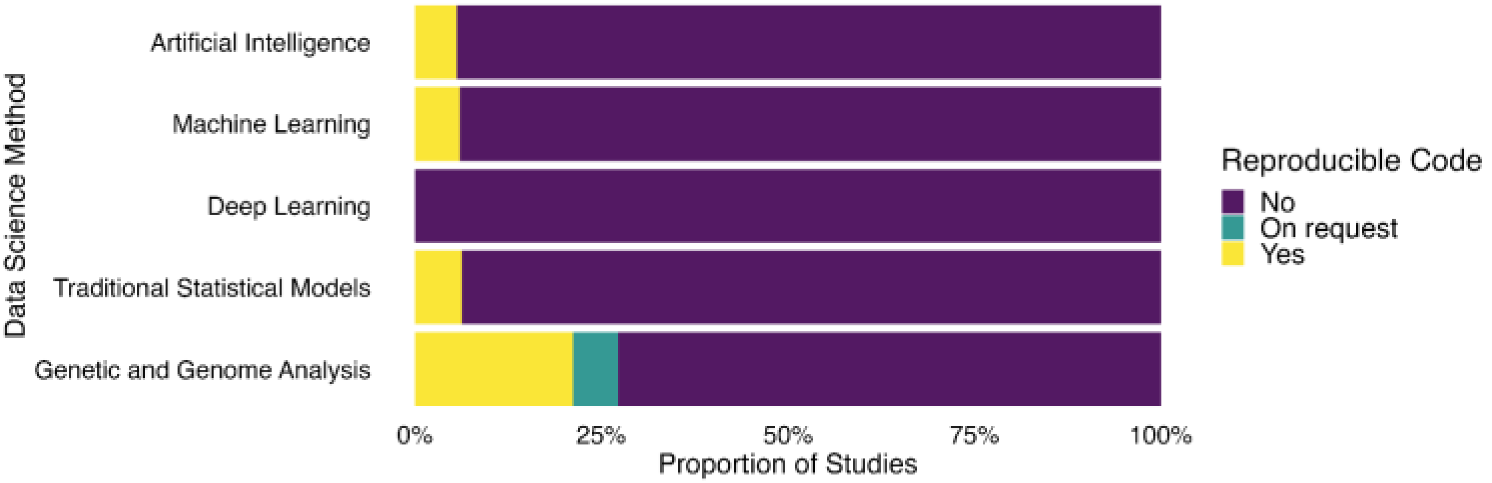

(a)

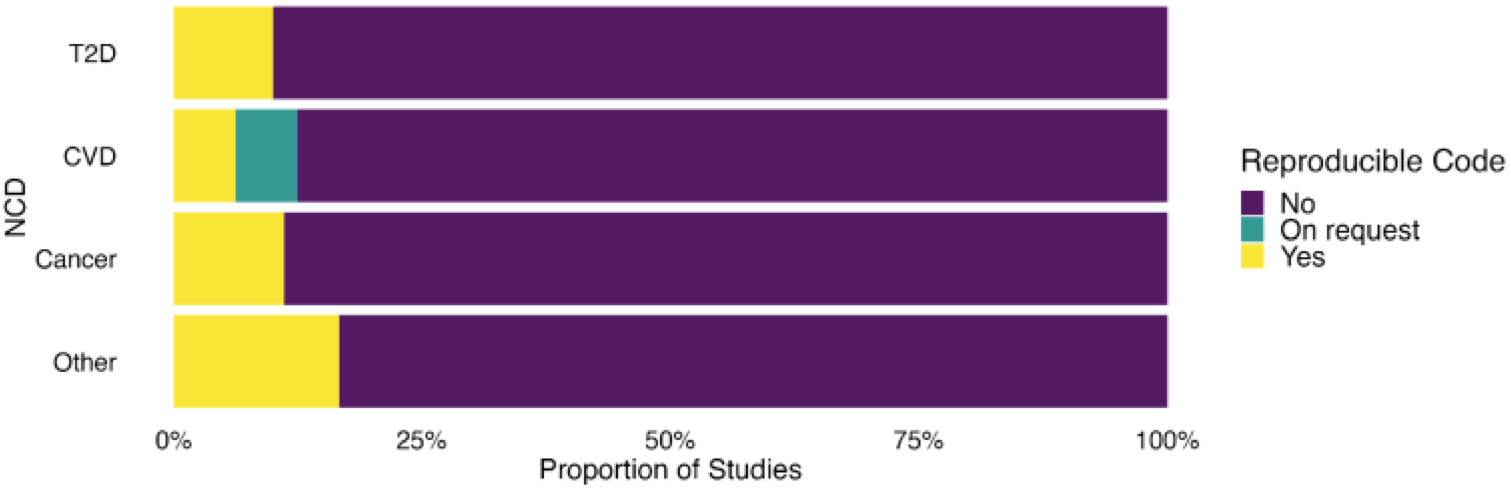

(b)

Figure S4: Distribution of open-source availability of the codes by Data Science Methods (a) and NCD (b) categories across countries in Africa

Supplementary Information References

1 Office of Data Science Strategy National Institute of Health. *NIH Strategic Plan for Data Science*, <https://datascience.nih.gov/sites/default/files/NIH_Strategic_Plan_for_Data_Science_Final_508.pdf> (2021).

2 Sheikh, H., Prins, C. & Schrijvers, E. in *Mission AI: The New System Technology* (eds Haroon Sheikh, Corien Prins, & Erik Schrijvers) 15-41 (Springer International Publishing, 2023).

3 Kaplan, J. *Defining Artificial Intelligence: What Everyone Needs to Know*. (Oxford University Press, 2016).

4 Owolabi, M. O. *et al.* Global synergistic actions to improve brain health for human development. *Nature Reviews Neurology* **19**, 371-383 (2023). https://doi.org:10.1038/s41582-023-00808-z

5 Thapa, R., Zengin, A. & Thrift, A. G. Continuum of care approach for managing non-communicable diseases in low- and middle-income countries. *J Glob Health* **10**, 010337 (2020). https://doi.org:10.7189/jogh.10.010337

6 LeCun, Y., Bengio, Y. & Hinton, G. Deep learning. *Nature* **521**, 436-444 (2015). https://doi.org:10.1038/nature14539

7 Mohri, M., Rostamizadeh, A. & Talwalkar, A. *Foundations of Machine Learning*. (The MIT Press, 2012).

8 França, R. P., Borges Monteiro, A. C., Arthur, R. & Iano, Y. in *Trends in Deep Learning Methodologies* (eds Vincenzo Piuri, Sandeep Raj, Angelo Genovese, & Rajshree Srivastava) 63-87 (Academic Press, 2021).

9 Vapnik, V. *The Nature of Statistical Learning Theory*. (Springer: New York, 2000).

10 Uffelmann, E. *et al.* Genome-wide association studies. *Nature Reviews Methods Primers* **1**, 59 (2021). https://doi.org:10.1038/s43586-021-00056-9

11 Sanderson, E. *et al.* Mendelian randomization. *Nature Reviews Methods Primers* **2**, 6 (2022). https://doi.org:10.1038/s43586-021-00092-5

12 Choi, S. W., Mak, T. S.-H. & O'Reilly, P. F. Tutorial: a guide to performing polygenic risk score analyses. *Nat. Protoc.* **15**, 2759-2772 (2020). https://doi.org:10.1038/s41596-020-0353-1

13 Fasman, K. H. & Salzberg, S. L. in *New Compr. Biochem.* Vol. 32 (eds Steven L. Salzberg, David B. Searls, & Simon Kasif) 29-42 (Elsevier, 1998).

14 World Health Organization (WHO). Noncommunicable diseases. (2023). https://doi.org:https://www.who.int/news-room/fact-sheets/detail/noncommunicable-diseases

15 Vos, T. *et al.* Global burden of 369 diseases and injuries in 204 countries and territories, 1990-2019: a systematic analysis for the Global Burden of Disease Study 2019. *The Lancet* **396**, 1204-1222 (2020). https://doi.org:10.1016/S0140-6736(20)30925-9

16 Gouda, H. N. *et al.* Burden of non-communicable diseases in sub-Saharan Africa, 1990-2017: results from the Global Burden of Disease Study 2017. *The Lancet Global Health* **7**, e1375-e1387 (2019). https://doi.org:10.1016/S2214-109X(19)30374-2

17 Boutayeb, A. in *Handbook of Disease Burdens and Quality of Life Measures* (eds Victor R. Preedy & Ronald R. Watson) 531-546 (Springer New York, 2010).

18 Schwartz, L. N., Shaffer, J. D. & Bukhman, G. The origins of the 4 × 4 framework for noncommunicable disease at the World Health Organization. *SSM - Population Health* **13**, 100731 (2021). https://doi.org:https://doi.org/10.1016/j.ssmph.2021.100731

19 Africa Centres for Disease Control and Prevention. Africa CDC Non Communicable Diseases, Injuries Prevention and Control and Mental Health Promotion Strategy

(2022-26). 34 (Africa Centres for Disease Control and Prevention, Addis Ababa, Ethiopia, 2022).
20 NCD Alliance. *NCDs: Noncommunicable diseases (NCDs) – mainly cancer, cardiovascular disease, chronic respiratory diseases, and diabetes – are the #1 cause of death and disability worldwide.*, <https://ncdalliance.org/why-ncds/NCDs> (2022).
21 Keates, A. K., Mocumbi, A. O., Ntsekhe, M., Sliwa, K. & Stewart, S. Cardiovascular disease in Africa: epidemiological profile and challenges. *Nature Reviews Cardiology* **14**, 273-293 (2017). https://doi.org:10.1038/nrcardio.2017.19
22 Roth, G. A. *et al.* Global Burden of Cardiovascular Diseases and Risk Factors, 1990–2019: Update From the GBD 2019 Study. *J. Am. Coll. Cardiol.* **76**, 2982-3021 (2020). https://doi.org:https://doi.org/10.1016/j.jacc.2020.11.010
23 Amegah, A. K. Tackling the Growing Burden of Cardiovascular Diseases in Sub-Saharan Africa. *Circulation* **138**, 2449-2451 (2018). https://doi.org:doi:10.1161/CIRCULATIONAHA.118.037367
24 Mendis, S. *et al. Global Atlas on cardiovascular disease prevention and control*. Published by the World Health Organization in collaboration with the World Heart Federation and the World Stroke Organization (2011).
25 Jamison, D. T. *et al.* Chapter 53. Public Health Surveillance: A Tool for Targeting and Monitoring Interventions. (2006).
26 Wagner, C. *et al.* Life course epidemiology and public health. *The Lancet Public Health* **9**, e261-e269 (2024). https://doi.org:10.1016/S2468-2667(24)00018-5
27 Janes, H., Pepe, M. S. & Gu, W. Assessing the value of risk predictions by using risk stratification tables. *Ann. Intern. Med.* **149**, 751-760 (2008). https://doi.org:10.7326/0003-4819-149-10-200811180-00009
28 Speechley, M. *et al.* Screening in Public Health and Clinical Care: Similarities and Differences in Definitions, Types, and Aims - A Systematic Review. *J Clin Diagn Res* **11**, Le01-le04 (2017). https://doi.org:10.7860/jcdr/2017/24811.9419
29 Wilson, J. M. G., Jungner, G. & World Health, O. *Public health papers ; no. 34* (World Health Organization, Geneva, 1968).
30 Califf, R. M. Biomarker definitions and their applications. *Experimental Biology and Medicine* **243**, 213-221 (2018). https://doi.org:10.1177/1535370217750088
31 Mayeux, R. Biomarkers: Potential uses and limitations. *NeuroRx* **1**, 182-188 (2004). https://doi.org:10.1602/neurorx.1.2.182
32 Bernabé-Ortiz, A. *et al.* Diagnostics and monitoring tools for noncommunicable diseases: a missing component in the global response. *Globalization and Health* **17**, 26 (2021). https://doi.org:10.1186/s12992-021-00676-6
33 Löwe, B. *et al.* Persistent physical symptoms: definition, genesis, and management. *The Lancet* **403**, 2649-2662 (2024). https://doi.org:10.1016/S0140-6736(24)00623-8
34 Hansebout, R. R., Cornacchi, S. D., Haines, T. & Goldsmith, C. H. How to use an article about prognosis. *Can. J. Surg.* **52**, 328-336 (2009).
35 Momsen, A.-M., Rasmussen, J. O., Vinther Nielsen, C., Iversen, M. D. & Lund, H. Multidisciplinary team care in rehabilitation: An overview of reviews. *J. Rehabil. Med.* **44**, 901-912 (2012). https://doi.org:10.2340/16501977-1040
36 Kolar, P. *Clinical rehabilitation*. (Alena Kobesová, 2014).
37 Wang, C. *et al.* Point-of-care diagnostics for infectious diseases: From methods to devices. *Nano Today* **37**, 101092 (2021). https://doi.org:https://doi.org/10.1016/j.nantod.2021.101092
38 Warren, A. D., Kwong, G. A., Wood, D. K., Lin, K. Y. & Bhatia, S. N. Point-of-care diagnostics for noncommunicable diseases using synthetic urinary biomarkers and

paper microfluidics. *Proceedings of the National Academy of Sciences* **111**, 3671-3676 (2014). https://doi.org:doi:10.1073/pnas.1314651111

39 King, K. R. *et al.* Point-of-Care Technologies for Precision Cardiovascular Care and Clinical Research. *JACC: Basic to Translational Science* **1**, 73-86 (2016). https://doi.org:doi:10.1016/j.jacbts.2016.01.008

40 United Nations. *Definition of major areas and regions*, <http://esa.un.org/unpp/definition.html> (
