# Supplementary material for: Harnessing data science to control non-communicable diseases in Africa: a systematic review and gap analysis
Source: Commun Med (Lond). 2026 Jul 16;6:397. doi: 10.1038/s43856-025-01272-0 (PMC13377181; doi:10.1038/s43856-025-01272-0)
Supplement: Supplementary file 2 — Description of Additional Supplementary files [file 43856_2025_1272_MOESM2_ESM.docx]

**Description of Additional Supplementary Files**

Supplementary Data 1: Source Data for Figures 2.

Supplementary Data S2: Preliminary search strategy and execution 3.

Supplementary Data S3: A summary of characteristics of included studies on Data Science methods for noncommunicable disease prevention and control in Africa 4.

Supplementary Data S4: Data quality, development, validation, performance and utility applied in Data Science methods for non-communicable disease prevention and control in Africa 5.

Supplementary Data S5: Pragmatic Recommendations for Tackling Gaps to Harnessing Data Science for NCD Prevention and Control in Africa
